# Supplementary material for: Can ICT investment promote green development? new insights from highly polluting listed enterprises in China
Source: PLoS One. 2024 Dec 30;19(12):e0316337. doi: 10.1371/journal.pone.0316337 (PMC11684712; doi:10.1371/journal.pone.0316337)
Supplement: Attachment — Submitted filename: Response to Reviewers.docx [file pone.0316337.s003.docx]

Response letter

**Title: Can ICT investment promote green development? New insights from highly polluting listed enterprises in China**

**Number.: PONE-D-24-34856**

We express our profound gratitude to the esteemed editors and two diligent reviewers for their invaluable contributions in the form of constructive comments and suggestions. Their insightful feedback has played a pivotal role in improving the overall quality and scholarly merit of our manuscript. We have meticulously addressed each comment and suggestion in a detailed and methodical manner, offering comprehensive responses in the section below. Additionally, for your easy reference, **all the main amendments in the revised manuscript are highlighted in red**. Furthermore, the comments and recommendations provided by the editors and reviewers have been distinctly presented in italics, enabling effortless comprehension and reference.

**Response to the Editor:**

**Editor point #1:** *The connection between ICT investment and green development, particularly in the context of highly polluting industries, needs further theoretical and empirical articulation. Reviewers noted a need for clearer explanations regarding hypotheses and endogeneity concerns.*

**Author response #1:** Thank you for your constructive comments, we make the following instructions on the modification of this issue. (1) This paper further discusses the relationship between ICT investment and green development of enterprises in high-pollution industries. This paper first discusses the environmental pressure, regulatory pressure, social responsibility and public opinion challenges faced by high-pollution industries on the road of green development, which enhances the rationality of this study (Section 1, page 4-5). Secondly, we expand the discussion of the unique advantages of ICT investment in promoting the green development of enterprises in high-pollution industries. It is discussed that ICT investment helps enterprises to realize intelligent monitoring and management of production process, realize information sharing and collaboration of enterprises in supply chain, and better carry out green technology R&D cooperation with stakeholders, so as to promote energy conservation, emission reduction and green development of enterprises (Section 1, page 6). (2) According to the reviewer's suggestion, we modify Hypothesis 2 and Hypothesis 3 to make their expressions clearer (Section 3.2.1 and 3.2.2, page 17,18). (3) We also further explain the exogeneity of the first mechanism test method in this paper, and delete the second mechanism test method that may have endogenous problems (Section 4.1.2, page 19). (4) We further explain the exogeneity of the first instrumental variable, and also delete the second instrumental variable that may have endogenous problems (Section 5.2.1, page 28-29). (5) We add the measurement method of the independent variables in this paper and add the results to the robustness test section (Section 5.2.2, page 31).

**Editor point #2:** *The integration of network effect theory into the analysis requires more depth. Additionally, addressing competing or complementary theories will strengthen the paper's contribution to the literature.*

**Author response #2:** Thank you for your constructive comments, we make the following instructions on the modification of this issue. (1) In the theoretical framework, we put the network effect theory into the analysis more deeply. First of all, we discuss how ICT investment affects green development at the enterprise level through network effects. Subsequently, we supplement the role of production equipment network, internal department network and online sales network constructed by enterprises through ICT investment, highlighting their impact on the green development of enterprises. Finally, we discuss the role of internal network platform and external network platform in promoting the green development of enterprises (Section 3.1, page 12-13). (2) We supplement the theory that may explain the relationship between ICT investment and corporate green development, and specifically combine the innovation diffusion theory or resource-based view to analyze the impact of ICT investment on corporate green development (Section 3.1, page 13-14).

**Response to Reviewer 1:**

**Reviewer point #1:** *The background could provide more specific industry-level challenges related to green development in high-pollution sectors. More emphasis on the environmental and regulatory pressures these industries face would strengthen the case for the study. The problem statement lacks clarity in terms of how ICT investment specifically addresses these environmental challenges compared to other corporate investments. Further exploration of why ICT investment is uniquely impactful would make the problem more compelling.*

**Author response #1:** Thank you for your valuable feedback and constructive suggestions on our manuscript. (1) In terms of background, we provide the specific industry-level challenges faced by enterprises in high-pollution industries in terms of green development. We mainly discuss the environmental pressure, regulatory pressure, social responsibility and public opinion challenges faced by enterprises in high-pollution industries (Section 1, page 4-5). (2) We increase the number of words on the unique advantages of ICT investment in promoting the green development of enterprises in high-pollution industries. It is discussed that ICT investment helps enterprises to realize intelligent monitoring and management of production process, realize information sharing and collaboration of enterprises in supply chain, and better carry out green technology R&D cooperation with stakeholders, so as to promote energy conservation, emission reduction and green development of enterprises (Section 1, page 6).

**Reviewer point #2:** *The contributions section could be more clearly articulated. While the paper mentions filling a gap, it does not sufficiently specify how its findings advance theoretical understanding or provide unique empirical evidence. The practical contributions to managers or policymakers are underdeveloped, limiting the broader relevance of the findings.*

**Author response #2:** Thank you for your insightful feedback on our manuscript. We have expanded the contribution part of the article to make the theoretical and practical significance of the article clearer. The specific expansion part is as follows: (1) Increase the number of words in the theoretical significance of this paper to study the relationship between ICT investment and green development from the enterprise level. (2) Increase the number of words in the theoretical significance of this paper to study the relationship between ICT investment and corporate green development from the perspective of non-linear relationship. (3) Increase the number of words about the actual contribution of the conclusions of this study to managers or decision makers (Section 1, page 7-8).

**Reviewer point #3:** *The theoretical framework is underdeveloped. While network effect theory is mentioned, it is not thoroughly integrated throughout the analysis. The paper could do more to show how the theory connects ICT investment to firm-level green outcomes. There is limited discussion of competing or complementary theories that might also explain the relationship between ICT and green innovation, such as innovation diffusion theory or resource-based theory.*

**Author response #3:** Thank you for your constructive comments, we make the following instructions on the modification of this issue. (1) In the theoretical framework, we put the network effect theory into the analysis more deeply. First of all, we discuss how ICT investment affects green development at the enterprise level through network effects. Subsequently, we supplement the role of production equipment network, internal department network and online sales network constructed by enterprises through ICT investment, highlighting their impact on the green development of enterprises. Finally, we discuss the role of internal network platform and external network platform in promoting the green development of enterprises (Section 3.1, page 12-13). (2) We supplement the theory that may explain the relationship between ICT investment and corporate green development, and specifically combine the innovation diffusion theory or resource-based view to analyze the impact of ICT investment on corporate green development (Section 3.1, page 13-14).

**Reviewer point #4:** *The theoretical framework is underdeveloped. While network effect theory is mentioned, it is not thoroughly integrated throughout the analysis. The paper could do more to show how the theory connects ICT investment to firm-level green outcomes. There is limited discussion of competing or complementary theories that might also explain the relationship between ICT and green innovation, such as innovation diffusion theory or resource-based theory.*

**Author response #4:** Thank you for your constructive comments. This question is the same as question 3, and we have made the following changes. (1) In the theoretical framework, we put the network effect theory into the analysis more deeply. First of all, we discuss how ICT investment affects green development at the enterprise level through network effects. Subsequently, we supplement the role of production equipment network, internal department network and online sales network constructed by enterprises through ICT investment, highlighting their impact on the green development of enterprises. Finally, we discuss the role of internal network platform and external network platform in promoting the green development of enterprises (Section 3.1, page 12-13). (2) We supplement the theory that may explain the relationship between ICT investment and corporate green development, and specifically combine the innovation diffusion theory or resource-based view to analyze the impact of ICT investment on corporate green development (Section 3.1, page 13-14).

**Reviewer point #5:** *There is limited explanation of why certain high-pollution industries were selected or how they differ in terms of ICT adoption and green development potential. The methodological section does not sufficiently discuss potential biases or limitations in the data, such as the use of proxies for ICT investment or green development.*

**Author response #5:** Thank you for your constructive comments, we make the following instructions on the modification of this issue. (1) We discuss the outstanding advantages and great potential of enterprises in high-polluting industries in using ICT investment to achieve green development (Section 1, page 4-5; Section 4.1.4, page 20). (2) In the robustness test, we discuss the limitations of the measurement of dependent variable and independent variable in this paper, and continue to add other measurement methods of independent variable for robustness test. The main limitation of the dependent variable is that the carbon emission intensity reflects the emission of carbon dioxide pollutants in the enterprise, and it does not reflect the emission of other pollutants and waste treatment in the enterprise. The robustness test in this paper uses whether the enterprise is a key pollution monitoring enterprise, which can break through this limitation to a certain extent. Thank you for your constructive comments, we will continue to learn other important measurement methods of the green development level of enterprises in the future research. The limitation of the independent variable is that the three types of ICT capital stock reflect the absolute level of ICT investment, and the relative level of ICT investment is insufficient. Therefore, in addition to the previously used degree of digital transformation of enterprises, the robustness test of independent variable also increases the relative level of ICT investment of enterprises, that is, the proportion of ICT investment of enterprises in total assets of enterprises. We add this result to the robustness test (Section 5.2.2, page 30-31).

**Reviewer point #6:** *There is limited explanation of why certain high-pollution industries were selected or how they differ in terms of ICT adoption and green development potential. The methodological section does not sufficiently discuss potential biases or limitations in the data, such as the use of proxies for ICT investment or green development.*

**Author response #6:** Thank you for your constructive comments. This question is the same as question 5, and we have made the following changes. (1) We discuss the outstanding advantages and great potential of enterprises in high-polluting industries in using ICT investment to achieve green development (Section 1, page 4-5; Section 4.1.4, page 20). (2) In the robustness test, we discuss the limitations of the measurement of dependent variable and independent variable in this paper, and continue to add other measurement methods of independent variable for robustness test. The main limitation of the dependent variable is that the carbon emission intensity reflects the emission of carbon dioxide pollutants in the enterprise, and it does not reflect the emission of other pollutants and waste treatment in the enterprise. The robustness test in this paper uses whether the enterprise is a key pollution monitoring enterprise, which can break through this limitation to a certain extent. Thank you for your constructive comments, we will continue to learn other important measurement methods of the green development level of enterprises in the future research. The limitation of the independent variable is that the three types of ICT capital stock reflect the absolute level of ICT investment, and the relative level of ICT investment is insufficient. Therefore, in addition to the previously used degree of digital transformation of enterprises, the robustness test of independent variable also increases the relative level of ICT investment of enterprises, that is, the proportion of ICT investment of enterprises in total assets of enterprises. We add this result to the robustness test (Section 5.2.2, page 30-31).

**Reviewer point #7:** *The paper presents a well-structured analysis of how ICT investment influences green development in high-pollution industries, focusing on the role of green technological innovation. The dynamic panel threshold model and heterogeneity analysis add depth to the research. However, the theoretical framework, motivation, and policy implications need further development to fully realize the potential of the study.*

**Author response #7:** Thank you for your constructive comments, we make the following instructions on the modification of this issue. We first modify the theoretical framework of the article, discuss the impact of ICT investment on enterprise green development through network effects more deeply and accurately, and expand the supplementary theoretical analysis of the impact of ICT investment on enterprise green development (Section 3.1, page 12-14). Second, we discuss in detail the specific challenges faced by enterprises in high-pollution industries on the road of green development and their outstanding advantages in using ICT investment to achieve green development, so as to enhance the rationality of this study (Section 1, page 4-6). Finally, we discuss the marginal contribution of the article in detail, further highlighting the theoretical significance of this study and the practical significance to decision makers (Section 1, page 7-8). At the end of the article, we put forward specific policy recommendations to promote the green development of enterprises from the two levels of enterprises and government.

We express our sincere gratitude for your valuable and perceptive comments.

**Response to Reviewer 2:**

**Major Comments:**

**Reviewer point #1:** *Line 227: How the authors relate the "threshold effect in networks" with their hypothesized " threshold effect in ICT" needs more articulation or clarification.*

**Author response #1:** Thank you for your constructive comments, we make the following instructions on the modification of this issue. We supplement the threshold effect of ICT investment in the article (Section 3.1, page 15). The added content is: “ICT investment has an important impact on enterprise production, innovation and energy conservation and emission reduction behavior mainly through network effects, such as the enterprise production equipment network, the internal department network of the enterprise, and the network formed by the enterprise and external related entities mentioned above. As more production equipment within the enterprise is interconnected, more internal departments within the enterprise are interconnected, and enterprises are interconnected with more external stakeholders, enterprises can greatly improve production efficiency and green technology innovation level through ICT investment, thus achieving energy conservation, emission reduction and green development”.

**Reviewer point #2:** *Line 265: Hypothesis 2 needs paraphrasing. E.g., " Production efficiency is one mechanism through which ICT investment impacts on green development of enterprises in highly polluting industries". The same for hypothesis 3; the phrase "mechanism role" should be improved.*

**Author response #2:** Thank you for your constructive comments, we have modified it according to your suggestion (page 17,18). Hypothesis 2 is modified as: “Production efficiency is one mechanism through which ICT investment impacts on green development of enterprises in highly polluting industries”. Hypothesis 3 is modified as: “Green technological innovation is one mechanism through which ICT investment impacts on green development of enterprises in highly polluting industries”.

**Reviewer point #3:** *Line 308: What the source of this potential endogeneity is, and how testing only the influence of the independent variable avoids this endogeneity needs clarification.*

**Author response #3:** Thank you for your thoughtful comments on our manuscript. The second mechanism test method used before in this paper is to add the mechanism variable to the benchmark regression, and then observe whether the size of the independent variable coefficient has changed significantly compared with the results before the mechanism variable are added. The disadvantage of this method is that the mechanism variable is a pre-treatment variable, which occurs after the independent variable. There may be an unobservable variable that affects both the mechanism variable and dependent variable, which leads to a significant endogenous problem in the regression model (Angrist & Pischke, 2009). Therefore, this method of adding mechanism variable to the benchmark regression model is less used in the field of economic research. In most cases, studying the influence of independent variables on dependent variables and the influence of independent variables on mechanism variables can already realize the mechanism test (Dell, 2010). Considering the possible endogenous problems, we finally delete the second mechanism test method (Section 4.1.2, page 19).

**Reviewer point #4:** *Line 311: Can the authors find a statistical/econometric means to establish this influence rather than depending merely on common sense?*

**Author response #4:** Thank you for your thorough review and valuable feedback. For observational data research, the real data generation process is complicated. It is not easy to find a suitable research situation to study the causal relationship between independent variables and dependent variables, and it is difficult to study the mediating effect (adding mechanism variables to the benchmark model). Therefore, the study of the mediating effect may be more suitable for the study as a topic alone. Of course, we also note that there is another structural approach empirical research paradigm in economics. The advantage of this paradigm is that the mediating effect analysis can be carried out more strictly, but the disadvantage is that the construction of the model often needs to rely on strong assumptions about the function form or the distribution form of unobservable variables (Heckman et al., 2013). As a research paradigm different from the empirical part of this paper, structuralism will be discussed in the later study, so that our analysis of the mechanism will be more rigorous.

**Reviewer point #5:** *Line 314: What is ICT shows no significant effect on MNV in model (2). Have the authors checked this before they use MNV as a control variable in Model (3), or they think it does not matter?*

**Author response #5:** Thank you for your constructive comments. The regression results of Model (2) show that ICT investment has a significant role in promoting the two mechanism variables (*MNV*) production efficiency (Ln*TFP*) and green technology innovation (Ln*GI*). The regression results are shown in Table 7. In addition, considering the possible endogenous problems, we delete the previous Model (3).

**Reviewer point #6:** *Line 317: The authors need a reference to substantiate this claim.*

**Author response #6:** Thank you for your constructive comments. Considering the possible endogenous problems, we finally delete the previous Model (3) and related viewpoints. The literature on the positive impact of production efficiency on the green development of enterprises can be seen in Section 3.2.1 and Reference No.39 of the paper. The literature on the positive impact of green technology innovation on the green development of enterprises can be seen in Section 3.2.2 and Reference No.41 of the paper.

**Reviewer point #7:** *L 370: This acronym comes all of a sudden. The full phrase should be introduced for the first time, with the acronym in the brackets.*

**Author response #7:** Thank you for your valuable feedback. The first “LP” has been modified to “Levinsohn-Petrin”, and “LP” is placed in parentheses (Section 4.2.3, page 22).

**Reviewer point #8:** *L377: The claim "The greater LnPgdp, the stronger the environmental awareness and green consumption ability of local citizens" needs to be supported by a reference.*

**Author response #8:** Thank you for your constructive comments. Considering your proposal to reduce the length of the empirical part, we deleted the analysis of the influence of control variables on the dependent variables (Section 4.2.4, page 23). It is also because this part is not very important to the influence of the independent variables on the dependent variables. However, we still found the references and put them at the end of this document (Dong et al., 2018).

**Reviewer point #9:** *L 381: In the sentence "During most of the sample period, the efficiency of resource development and utilization in most provinces in China has been high, so Rse is good for green development of local enterprises" how the first phrase of this sentence leads to the second (so...) is not clear. I mean does the first phrase automatically lead the conclusion that "Rse is good for green development"?*

**Author response #9:** Thank you for your thorough review and valuable feedback. Considering your proposal to reduce the length of the empirical part, we deleted the analysis of the influence of control variables on dependent variables (Section 4.2.4, page 23). There is indeed a jump in the previous writing logic, and we reinterpret it here in response to the comments. What we want to express is that in the case of high resource development and utilization efficiency in the province, the more prominent resource endowment of the province will become an advantage for the green development of local enterprises. This is because companies in the province can use the natural resources of the region to achieve a rapid increase in economic profits, and because of mature resource extraction and processing technology, natural resources do not generate too much carbon dioxide in the process of creating economic value. On the contrary, in the case of low efficiency of resource development and utilization in provinces, the more prominent resource endowment of provinces will become a disadvantage for the green development of local enterprises, because enterprises will produce too much carbon dioxide in the exploitation and utilization of resources (Zhang et al., 2022).

**Reviewer point #10:** *L 398: Why +1? Are there enterprises with 0 employees?*

**Author response #10:** Thank you for your thorough review and valuable feedback. Here is the author's pen error, and we are actually the number of employees directly logarithm. Now the modification has been completed (Section 4.2.4, page 23). In this paper, there is no enterprise with 0 employees, because the abnormal samples with less than or equal to 0 employees have been eliminated in the sample processing stage. From the descriptive statistical results of the variables in this paper, it can also be seen that the minimum value of the enterprise scale (measured by the logarithm of the number of employees) is 4.956.

**Reviewer point #11:** *L 398: The sentence "Large-size enterprises have stronger environmental awareness" needs a reference.*

**Author response #11:** Thank you for your constructive comments. Considering your proposal to reduce the length of the empirical part, we deleted the analysis of the influence of control variables on dependent variables (Section 4.2.4, page 23). However, we still found the references and put them at the end of this document (Li et al., 2019).

**Reviewer point #12:** *L 475: It would be better not to mix models with the results section. All models and their explanation may better be dealt with in section 4, and dedicate section 5 to results and discussion. Moreover, these two sections are very lengthy; they need to be shortened significantly. That way, the paper will be easier to follow.*

**Author response #12:** Thank you for your thorough review and valuable feedback. Based on your suggestion, we put the multi-period DID model and the parallel trend test model of the results section in section 4, and devoted section 5 to the analysis and discussion of empirical results (Section 4.1.3, page 19-20). At the same time, we have refined the section 4 and section 5 of this article, and deleted the relatively less important text description and the substitution regression method in the robustness test, so as to make the empirical part of this article clearer and easier to understand.

**Reviewer point #13:** *L 502: The authors write "... the carbon emission intensity of an enterprise is difficult to affect the topographic condition of the city where it is located." In relation to the validity of an IV, this sentence should read the other way around, i.e., "the topographic condition of the city is difficult to affect the carbon emission intensity of an enterprise". Moreover, the authors need to elaborate on why they think so.*

**Author response #13:** Thank you for your constructive comments, we make the following instructions on the modification of this issue. From the perspective of exogenous conditions, it is indeed necessary to explain that the terrain conditions of the city are difficult to directly affect the carbon emission intensity of enterprises. We modified the original text and re-discussed the exogeneity of the variable (Section 5.2.1, page 28-29).

**Reviewer point #14:** *L 506: The authors write "the carbon emission intensity of enterprises cannot directly affect the change of the ratio of ICT assets to total assets of enterprises." The cause and effect relationship should be reversed. The validity of the IV should also be better motivated. Just writing "...cannot directly affect..." is not enough to convince readers that your IV is valid.*

**Author response #14:** Thank you for your thorough review and valuable feedback. In terms of exogeneity, we should indeed show that the proportion of ICT assets in total assets will not directly affect the carbon emission intensity of enterprises. However, considering that the exogenous nature of this instrumental variable may not be enough, we finally deleted the instrumental variable of the proportion of enterprise ICT assets to total assets. The reason for the lack of exogeneity is that if there is a reverse causal relationship between the carbon emission intensity of enterprises and the level of ICT investment of enterprises, the proportion of ICT assets in total assets of enterprises will still have a strong relationship with the carbon emission intensity of enterprises, that is to say, this instrumental variable cannot well alleviate the endogenous problem of reverse causality. In contrast, the topographic relief variable can better alleviate the endogenous problem of reverse causality, because the topographic relief as a natural variable will not change with the change of enterprise carbon emission intensity.

**Reviewer point #15:** *L 562: Have the authors conducted a statistical test to show that the two values (0.0432 and 0.0504) are significantly different?*

**Author response #15:** Thank you for your constructive comments. If we continue to use the previous mediation effect model (3), it is indeed necessary to test the coefficient difference between the two. However, considering the possible endogenous problems of the previous Model (3), we delete the previous Model (3) and its related conclusions.

**Reviewer point #16:** *L 573: The same comment as above.*

**Author response #16:** Thank you for your constructive comments. Consistent with the response to the above question, if we continue to use the previous mediation effect model (3), it is indeed necessary to test the coefficient difference between the two. However, considering the possible endogenous problems of the previous Model (3), we delete the previous Model (3) and its related conclusions.

**Minor Comments:**

**Reviewer point #1:** *1. Line 93: The phrase "cooperation between enterprises and other enterprises" is ambiguous.*

**Author response #1:** Thank you for your constructive comments, we make the following instructions on the modification of this issue. It has been changed to: “the efficiency of cooperation between enterprises and its supply chain upstream and downstream enterprises is low”.

**Reviewer point #2:** *Line 112 and elsewhere: The word "literatures" should be replaced with the singular "literature".*

**Author response #2:** Thank you for your thorough review and valuable feedback. All the words “literatures” in this article have been changed into singular form.

**Reviewer point #3:** *Line 364: This acronym comes all of a sudden. The full phrase should be introduced for the first time, with the acronym in the brackets.*

**Author response #3:** Thank you for your constructive comments. The first “LP” has been modified to “Levinsohn-Petrin”, and “LP” is placed in parentheses.

Once again, we sincerely appreciate your insightful comments and your role in improving our manuscript.

**References mentioned in the reply:**

[1] Angrist, J. D., & Pischke, J. S. (2009). Mostly harmless econometrics: An empiricist's companion. Princeton university press.
[2] Dell, M. (2010). The persistent effects of Peru's mining mita. Econometrica, 78(6), 1863-1903.
[3] Heckman, J., Pinto, R., & Savelyev, P. (2013). Understanding the mechanisms through which an influential early childhood program boosted adult outcomes. American Economic Review, 103(6), 2052-2086.
[4] Dong, F., Yu, B., Hadachin, T., Dai, Y., Wang, Y., Zhang, S., & Long, R. (2018). Drivers of carbon emission intensity change in China. Resources, Conservation and Recycling, 129, 187-201.
[5] Zhang, M., Yan, T., & Ren, Q. (2022). Does innovative development drive green economic growth in resource-based cities? Evidence from China. Frontiers in Environmental Science, 9, 745498.
[6] Li, G., Wang, X., Su, S., & Su, Y. (2019). How green technological innovation ability influences enterprise competitiveness. Technology in Society, 59, 101136.

We would like to express our sincere gratitude for your insightful comments and your valuable contribution towards enhancing the quality of our manuscript. We have made diligent efforts to enhance our manuscript and have implemented several changes accordingly. It is important to note that these modifications have been made without altering the underlying content of our manuscript.

We extend our heartfelt gratitude to the editor and the two reviewers for their dedicated and diligent efforts in evaluating our manuscript. We earnestly hope that the revisions we have made will meet their expectations and receive their approval.

Once again, thank you very much for your valuable comments and suggestions.

Yours faithfully

Qingbo Li
